# Reduced expression of the electron transport chain component *ATPsynβL* in glutamate neurons changes *Drosophila melanogaster* sleep patterns through adulthood.

**DOI:** 10.17912/micropub.biology.002033

**Published:** 2026-02-27

**Authors:** Abigail Forrest, Maria Longenecker, Marciella V. Shallomita, Elaine Miranda Perez, Savanna Hinson, Jay Hirsh, B. Jill Venton, Jeffrey M. Copeland

**Affiliations:** 1 Eastern Mennonite University, Harrisonburg, VA, US; 2 University of Virginia, Charlottesville, VA, US; 3 Biology, University of Virginia, Charlottesville, VA, US; 4 Chemistry, University of Virginia, Charlottesville, VA, US; 5 Biology, Eastern Mennonite University, Harrisonburg, VA, US

## Abstract

A physiological marker of human aging is a decline in sleep patterns, a behavior also found in *Drosophila melanogaster* . To understand the connection between aging and sleep, we monitored sleep in long-lived flies. RNAi targeting the electron transport chain *ATPsynβL* gene in glutamate neurons has been demonstrated to extend life span. We investigate the sleep behavior in these RNAi flies at 5 days and 30 days of age and observe a persistent increase in daytime sleep, but not in nighttime sleep or sleep bout length. These results demonstrate the unique effects on sleep by glutamate-specific RNAi of *ATPsynβL* .

**Figure 1. Effects of sleep in 5 and 30 day old flies with RNAi targeting ATPsynβL in glutamate neurons:**
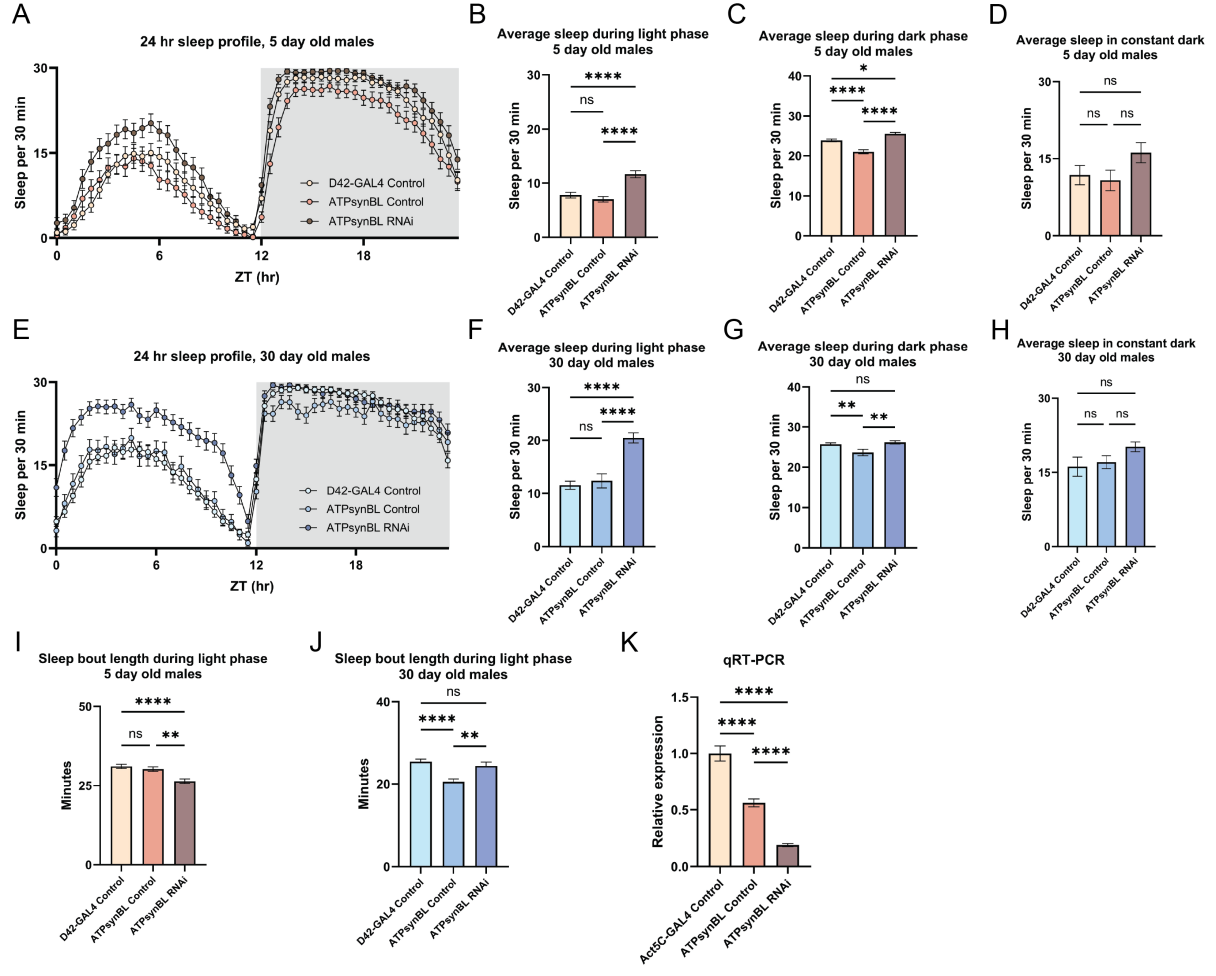
The panels show the effects on sleep at 5 days and 30 days of age in flies with glutamate neuron-specific RNAi of the *ATPsynβL* gene. The GAL4 control strain is heterozygous D42-GAL4 (A-J) or Act5C-GAL4 (K). The *ATPsyn* β *L* control represents heterozygous flies with inactivated UAS- *ATPsyn* β *L* -RNAi, while the *ATPsyn* β *L* RNAi line carries both GAL4 and UAS- *ATPsyn* β *L* -RNAi elements. (A-D) The average amount of sleep time for 5 day old flies. (A) The 24 hour profile of an average sleep amount for three days in light:dark (LD) conditions. (B-C) The average amount of sleep during the light phase and dark phase of an LD cycle. (D) The average amount of sleep once the flies are in 24 hours of darkness. (E-H) The average amount of time asleep for 30 day old flies. (E) The 24 hour profile of an average sleep amount for three days in LD conditions. (F-G) The average amount of sleep during the light phase and dark phase of an LD cycle. (H) The average amount of sleep in 24 hours of darkness. (I-J) The average sleep bout length during the light phase of a 24 hour LD cycle in 5 day old flies and 30 day old flies. (K) Quantitative real-time PCR for flies with ubiquitous knockdown of *ATPsyn* β *L* . P values of <.05 (*), <.01 (**), <.001 (***), <0.0001 (****), and not significant (ns) are indicated. Error bars show S.E.M. ranges.

## Description

Aging is a universal process that manifests with disrupted sleep patterns, declines in cognitive function, and increased tissue degeneration (Jagust, 2013). Mitochondrial activity and elongation are known determinants of aging, and RNAi targeting the electron transport chain has been shown to prolong life span and drive sleep induction (Sarnataro et al., 2024; Rana et al., 2017; Wang & Hekimi, 2015; Copeland et al., 2009). The first four complexes of the electron transport chain create a proton gradient across the inner mitochondrial membrane, while complex V couples proton movement to the production of ATP from ADP. In *Drosophila* , 13 proteins comprise complex V, and *ATPsynβL* is a gene duplication of the core *ATPsyn* β subunit (Tripoli et al., 2005). RNAi of *ATPsynβL* in different neuron subtypes has varied effects on *Drosophila* aging. Targeting RNAi of *ATPsynβL* to glutamate neurons is sufficient to extend life span, while targeting dopamine or serotonin neurons has no effects (Keppley et al., 2018; Landis et al., 2023).

Total sleep time is correlated with longevity, with individuals with insufficient amounts of sleep showing an increased risk of early mortality (Ungvari et al., 2025). In *Drosophila* , this correlation is commonly observed, though not universally true. For example, *sleepless* mutants exhibit an 80% reduction in sleep and have a truncated life span, but *fumin* flies sleep less and have a typical life span (Koh et al., 2008; Kume et al., 2005). *Drosophila melanogaster* , therefore, serves as a good model for discerning the particular neurological and genetic aspects that tether sleep and aging.

In this report, we investigated the connection between sleep and aging in flies with *ATPsynβL* RNAi activated in glutamate neurons (Landis et al., 2023). Using *Drosophila* activity monitors, we tracked the sleep behaviors of young (5 days old) and older (30 days old) males in a 24 hour light:dark (LD) cycle followed by 5 days in 24 hours of total darkness. In early adulthood, glutamate neuron-specific *ATPsynβL* knockdown showed a pronounced 50 – 66% increase in daytime sleep over the GAL4 and RNAi controls ( Figure 1A, B) but a more limited change (8 – 22%) during the nighttime phase ( Figure 1C ). After the switch to constant darkness, the activated RNAi flies display an increased tendency to sleep (37 – 50%), although the difference is not statistically significant.

Sleep amounts naturally change in flies during aging, but the *ATPsynβL* RNAi mutants retain the increased amount of daytime sleep, as 30 day old males sleep 65 – 77% more than the control strains ( Figure 1E, F). The increase in nighttime sleep, however, is not noticed in 30 day old flies, as *ATPsynβL* RNAi mutants sleep 11% more than the RNAi controls but sleep the same amount as the GAL4 control flies ( Figure 1G ). Similarly, 30 day old *ATPsynβL* RNAi flies sleep the same amount as the controls once transitioned to 24 hours of darkness. These results demonstrate that RNAi of *ATPsynβL* effects daytime but not nighttime sleep in young and older adult flies.

Measuring the length of sleep bouts can inform us about the possible fragmentation of sleep known to occur during aging. Since the age-related impact of sleep in the *ATPsynβL* RNAi mutants occurred only during the daytime phase, we were more interested in this time period. Interestingly, we observed consistent changes in sleep bout length in 5 day old activated RNAi flies but not in 30 day old flies. 5 day old *ATPsynβL* RNAi flies had sleep bouts 13 – 15% shorter than both controls, though 30 day old *ATPsynβL* RNAi flies had sleep bouts 14% longer than the RNAi controls and of equal length to the GAL4 controls ( Figure 1I, 1J). These results indicate that *ATPsynβL* RNAi has a consistent effect throughout adulthood on the total time asleep but not on sleep bout length.

To validate that the RNAi construct functioned to knockdown *ATPsynβL* expression, we conducted a qRT-PCR experiment but used the ubiquitous *Act5C* -GAL4 driver line. While the *Act5C* -GAL4 does not have the same pattern of GAL4 expression as the glutamate neuron *D42* -GAL4 line, the *Act5C* -GAL4 line offers one method to extract mRNA while still testing the effectiveness of RNAi. Measuring the effectiveness of RNAi in glutamate neurons using the original *D42* -GAL4 driver line would require using single-cell RNA sequencing or immunostaining with an untested anti- *ATPsynβL* antibody, both prohibitive procedures for the scope of these experiments. In our qRT-PCR experiments, we noticed a significant 80% knockdown of the *ATPsynβL* transcript from the GAL4 control line (P < 0.0001) ( Figure 1K ). The 40% knockdown of *ATPsynβL* in the RNAi control line could represent leaky expression of the UAS- *ATPsynβL* -RNAi construct and partial activation of RNAi. We note that despite the possible leakiness from the UAS- *ATPsynβL* -RNAi construct, the sleep behaviors of these RNAi control flies showed no difference in the time asleep during the daytime and constant darkness ( Figure 1B, D, F, H). During the dark phase of the 24 hour LD cycle, the UAS- *ATPsynβL* -RNAi&nbsp; controls slept less than the other strains, a result suggesting that the possible partial RNAi in these flies does not affect nighttime sleep behavior.

In this report, we investigated the possible connection between sleep and longevity, specifically in flies with glutamate neuron-specific RNAi targeting the complex V *ATPsynβL* gene. We observed that activated RNAi led to increases in total daytime sleep time at both 5 days and 30 days of age. Meanwhile, we did not notice any consistency in the amount of nighttime sleep, in constant darkness, or sleep bout length while these flies aged. While we have observed the persistent effect of increased daytime sleep, we unfortunately cannot tease out its importance in determining longevity. It is entirely possible that *ATPsynβL* RNAi in glutamate neurons could have independent effects on sleep and aging, rather than sleep directly affecting longevity. Previous works have demonstrated a role of glutamate neurons in promoting wakefulness and inhibiting sleep, though it is unresolved if the changes affect a particular phase of the LD cycle (Ly & Naidoo, 2019; Zimmerman et al., 2017). With these results in mind, it is entirely possible that the perturbations caused by the RNAi of *ATPsynβL* lead to the inactivation of glutamate neurons. It remains to be determined how glutamate neurons can affect sleep for a particular phase of a 24 hour cycle.

## Methods

**General husbandry**

Flies were fed standard molasses food and reared at 25°C with a 12-hour light:dark cycle. To minimize hybrid vigor in the studies, the RNAi line was backcrossed ten times to the *white ^1118^* laboratory strain and the *D42* -GAL4 strain twice (Dietzl et al., 2007). The GAL4 control strain and the RNAi control strain were the respective products of a cross between the *D42* -GAL4 and UAS- *ATPsynβL* -RNAi lines with the *white ^1118^* laboratory strain. The activated RNAi line was the result of a cross between the *D42* -GAL4 and the UAS- *ATPsynβL* -RNAi line.

**Activity monitor**

5 day old and 30 day old male flies were monitored for 3 days at 12:12 light:dark cycle, followed by 5 days of 24 hrs dark cycle with a 2 μW/cm2 intensity green LED light in Trikinetics activity monitors (Waltham, MA). Flies were maintained at 22˚C, 60% relative humidity in glass tubes with a plug of molasses food at one end. At least 60 male flies were used per condition.

**Quantitative real time PCR**

RNA from 5 day old flies was extracted using the RNeasy Mini protocol (Qiagen, Hilden, Germany), and isolated RNA was quantified using a NanoDrop spectrophotometer (Thermo Scientific, Wilmington, DE, USA). 700 micrograms of RNA were reverse transcribed using the iScript cDNA Synthesis kit (Bio-Rad, Hercules, CA). RT-qPCR was performed on a CFX Connect Detection System (Bio-Rad, Hercules, CA) using Sso Advanced Universal SYBR Green Mix (Bio-Rad, Hercules, CA).

Each sample was analyzed with six reactions, along with controls (NTCs) without cDNA in the PCR. The specificity of each amplified reaction was verified by a dissociation curve analysis after each measurement. To determine primer efficiency, serial 2-fold dilutions of each primer set were used to generate a standard curve, and efficiencies (E) were determined based on the slope (M) of the log−linear portion of the standard curve (E = 10−1/M − 1 × 100).

**Statistical analysis**

Data were analyzed using the ShinyR-DAM program (Cichewicz & Hirsh, 2018), GraphPad Prism (Version 10.5.0 (774), San Diego, CA) and Excel (Microsoft) software. One-way ANOVA tests (Tukey HSD) were run to determine statistical significance.

## Reagents

**Stocks**

The *D42* -GAL4 (RRID: BDSC_8816) and *Act5C* -GAL4 (RRID: BDSC_4414) fly strains were obtained from the Bloomington Stock Center (NIH P40OD018537, Bloomington, IN). The UAS- *ATPsynβL* -RNAi line (VDRC ID: 22112) targeting Complex V of the electron transport chain was purchased from the Vienna Drosophila RNAi Center (Vienna, Austria).&nbsp;

**Primers**

**Table:** 

Primer name	Targeted gene	DNA sequence
JC35	*Act5C* (FBgn0000042)	TTGTCTGGGCAAGAGGATCAG
JC36	*Act5C* (FBgn0000042)	ACCACTCGCACTTGCACTTTC
JC203	*ATPsynβL* (FBgn0036568)	AGGATGAAGCCGAGGATGAG
JC204	*ATPsynβL* (FBgn0036568)	GGAATACCTCCAGCACTAGGTTAGCA

The sequences for the Act5C DNA primers have been previously reported (Rana et al., 2017).
